# An Empirical Analysis Rejects the Hybrid Speciation Hypothesis of a Crucial Kiwifruit Species, Despite Genomic Evidence of Frequent Interspecific Gene Flow in the Genus

**DOI:** 10.3389/fgene.2019.01250

**Published:** 2020-02-04

**Authors:** Jie Yang, Weirui Fu, Haoming Xu, Zhiping Song, Wenju Zhang, Ji Yang, Yuguo Wang

**Affiliations:** Ministry of Education Key Laboratory for Biodiversity Science and Ecological Engineering, Institute of Biodiversity Science, School of Life Sciences, Fudan University, Shanghai, China

**Keywords:** kiwifruit, interspecific hybridization, *Actinidia fulvicoma*, hybrid speciation, species identification

## Abstract

Hybrid speciation is an important way to generate species diversity. In general, however, interspecific hybridization is easily confused with the formation of hybrid species. Using the genomic resequencing data of the kiwifruit genus (*Actinidia*), at least ten species were documented recently as homoploid hybrid species, and thus a two-layer mode of species diversification has been proposed. As a crucial piece of evidence, *Actinidia fulvicoma* was identified as a hybrid derivative of *Actinidia eriantha* × *Actinidia cylindrica*, representing a rare case of hybrid species in kiwifruit that won the competition of ecological niches with one of its putative parental species, *A. cylindrica*. However, the hypothesized hybrid origin of *A. fulvicoma* is inconsistent with our specimen observations. Here, we present multiple lines of evidence to reject the hybrid speciation hypothesis for this species, despite genomic evidence for frequent interspecific gene flow. We collected the samples of *A. fulvicoma* in type locality and neighboring regions to contrast them with type specimen, and sequenced nuclear ribosomal DNA ITS, chloroplast *trn*L-*trn*F and mitochondrial *nad2-i3*, as well as four single-copy nuclear genes explored from kiwifruit genomes, to infer phylogenetic relationships among *A. fulvicoma*, its putative parental species, and their relatives. Our data definitely reveal that *A. fulvicoma* occupies an independent backbone lineage and it is not a hybrid. This study suggests that correct evolutionary applications on extensive surveys of the putative hybrid and its possible parents with strict criteria are necessary in the documentation of hybrid speciation to advance our understanding of the genomic basis of hybrid species.

“Surprisingly, although there is widespread establishment of allopolyploid hybrids in *Actinidia*, homoploid hybrid speciation is prevalent in this genus... The hybrid *A. fulvicoma var. fulvicoma* should be a winner in the competition for ecological niches because one of its parents, *A. cylindrica*, has a very restricted distribution within its wide geographic range, suggesting that hybridization can widen ecological adaptation.” (Liu et al., 2017, p. 886)

## Introduction

As one of the important evolutionary forces, natural hybridization occurs frequently in flowering plants. It is estimated that an average of 25% of plant species are known to hybridize with at least one other species (Mallet, 2005). Interspecific hybrids from distantly related parent species are often sterile or inviable (Mayr, 1963; Coyne and Orr, 2004). Even if a healthy hybrid is formed, the offspring usually backcrosses to the more abundant parent species rather than going through self-fertilization, and will therefore often be unfit (Mallet, 2007). The fact that most plant hybrid zones are limited to an extent implies that hybrids are on average less fit than their parents, at least in parental habitats (Coyne, 1996; Rieseberg, 1997). However, in a few cases, the reproductive barrier between a few hybrid offspring and their parents were built up, and, eventually, the offspring could reproduce independently to form a new hybrid linage. For example, two diploid species of *Helianthus* (*H. annuus* and *H. petiolaris*) produced three diploid species (*H. anomalus*, *H. deserticola*, and *H. paradoxus*) through ancient hybridization (Rieseberg et al., 1995; Gross and Rieseberg, 2005). These hybrids are distributed in deserts, salt-rich swamps, or other suitable open habitats that are not occupied by parent species, respectively. Although interspecific hybridization in flowering plants is relatively common, tests of hybrid speciation hypotheses using phylogenetic approaches have rejected such hypotheses many times (e.g. Rieseberg and Wendel, 1993; Wolfe and Elisens, 1993; Wolfe and Elisens, 1994; Wolfe and Elisens, 1995; Arnold, 1997). Only a few cases of homoploid hybrid speciation have been documented when strict criteria are applied (Schumer et al., 2014). Despite the argument of whether these criteria are too stringent (Feliner et al., 2017; Schumer et al., 2018), there is no doubt that many putative hybrid species need to be re-tested.

Owing to frequent interspecific hybridization, the kiwifruit genus (*Actinidia* Lindl.) has been recognized as an ideal material for studying hybrid speciation. The distribution areas of wild kiwifruit species often overlap, and hybridization between taxa is common in nature (Huang and Liu, 2014). The *Actinidia* species are well documented for the contrasting mode of the maternal inheritance of mitochondrial (mt) genes and paternal inheritance of most chloroplast (cp) genes (Cipriani et al., 1995; Testolin and Cipriani, 1997; Chat et al., 1999; Li et al., 2013), and, like other flowering plants, their nuclear genes biparentally inherit. Through examining the phylogenetic incongruences among different kinds of genes such as nuclear ribosomal (nr) DNA ITS vs. cp gene *matK* (Li et al., 2002), mtDNA vs. cpDNA (Chat et al., 2004), and nuclear markers vs. cytoplasmic DNA sequences (Li et al., 2007), several taxa were identified as the putative interspecific hybrids in *Actinidia*.

Recently, the history of reticulate evolution of *Actinidia* was reconstructed through genomic DNA sequencing of 25 taxa, and the hybrid origins of at least ten species were documented (Liu et al., 2017). Based on these findings, a two-layer mode of species diversification was further proposed, and homoploid hybrid speciation was particularly emphasized as an important representative mechanism fueling rapid reticulate radiations. Unexpectedly, like *A. cylindrica* var. *reticulata*, *A. fulvicoma* var. *fulvicoma* was also documented as a hybrid species produced by *A. cylindrica* and *A. eriantha*, whereas the variety of this species, *A. fulvicoma* var. *hirsuta*, is not a hybrid (Liu et al., 2017). It is worth noting that the distribution of *A. cylindrica* is limited to a few counties in Guangxi, China, while *A. fulvicoma* var. *fulvicoma* is widely distributed in several provinces in southern China. They further concluded that the wide distribution of *A. fulvicoma* var. *fulvicoma* relative to *A. cylindrica* was the result of ecological differentiation promoted by hybridization.

Interspecific hybridization has proven to be the ecological differentiation of species (e.g., Rieseberg et al., 2003) and even the expansion of distribution areas of hybrids in some cases of homoploid hybrid speciation (Hovick and Whitney, 2014), but such special cases, like *A. fulvicoma* var. *fulvicoma* as mentioned above, are rare. Judging from voucher photographs of this species of Liu et al. (2017), however, it is inconsistent with our observations. The sample of *A. fulvicoma* of Liu et al. (2017) has pink flowers with seven petals, orange anthers, and gray fruit without persistent sepals. These characteristics do not completely match the original description of *A. fulvicoma*: white flower with five petals, yellow anthers, and dark green fruit with persistent sepals folded back. These morphological comparisons imply that it is likely that incorrect identification or a wrong sampling of *A. fulvicoma* led to error conclusion of its hybrid origin in the study of Liu et al. (2017).

To verify whether *A. fulvicoma* is of hybrid origin, we collected population samples from its type locality, Mount Luofu, Guangdong, and its adjacent areas. Phylogenetic analyses and mutation statistics based on multiple genes with different inherited modes, specimen comparisons involving type specimens and voucher photographs of Liu et al. (2017), and a geographic distribution analysis of *A. fulvicoma* and its putative parental species inferred by Liu et al. (2017) were produced to address whether interspecific hybridization occurred. However, our evidence does not support that *A. fulvicoma* is a hybrid species between *A. cylindrica* and *A. eriantha*.

## Materials and Methods

We present here a practical method to assess whether a species is of hybrid origin, and it involves DNA sampling, gene amplification, sequence analysis, and the comparison of distribution and morphology. We reason that if a taxon is not of hybrid origin, the samples collected from the type locality or neighboring areas would occupy independent lineages apart from putative parental species in both nuclear and cytoplasmic gene trees, or, if there exist more distinct variable mutations between the species and any putative parental species, it should not be a hybrid taxa. Similarly, in this case, the geographical distribution of the two putative parental species would not overlap. On the contrary, the nuclear genes of the hybrid species would contain genetic components of both parents, while the sequences of its cytoplasmic genes would be similar to those of one parent. In our notation, its distribution should be within, or not far from, the overlapping regions of two parent species.

### Sample Collection and DNA Extraction

The samples of *A. fulvicoma* and its varieties were collected from its type locality (Mount Luofu) and neighboring areas (Nanxiong and Mount Nankun) in Guangdong, China, and other localities such as Mount Daoyao and Nandan in Guangxi, China (**Table S1**). The two putative parental species (*A. eriantha* and *A. cylindrica*) and other *Actinidia* species were collected form the Guilin Botanical Garden, Guangxi, or from the field. All voucher specimens were deposited in the Herbarium of Fudan University (FUS). The species taxonomy primarily follows the classification of *Actinidia* in *Flora Republicae Popularis Sinicae* (i.e. the Chinese version of *Flora of China*, Liang et al., 1984).

The fresh young leaves were dried with silica gel and the total DNA of each sample was isolated using a Tiangen Plant Genomic DNA Kit (Tiangen Biotech Co., Beijing, China) following the manufacturer’s instructions. The quality of extracted genomic DNA was assessed by agarose gel electrophoresis.

### PCR Amplification and Gene Sequencing

The amplification and sequencing primers of the ITS gene were ITS4 and ITS5 (White et al., 1990). Primers c and f (Taberlet et al., 1991) were used as the amplification and sequencing primers of cpDNA *trn*L*-trn*F. The mtDNA *nad2-i3* sequences were amplified in two segments by the nested PCR method. Five primers (769F, 813F, 2F, 881R, and 1227R) were newly designed. The first set of primers were 1R (Cipriani et al., 2003), 769F (5'-GCGAAGAGAATAAGATGCTGC-3'), 2F (5'-TGAAATCACTGGTGCTCG-3'), and 1277R (5'-GCCTTTCCTTGAATGGTTG-3'). The first PCR amplification products were used as the DNA template for the second PCR. Primers 1R, 813F (5'-CCAGAATAAGATGCTGCTCC-3'), 2F, and 881R (5'-GCCTTAGAGGGGAAGATTAGC-3') were used as the second set of PCR primers. The primers 1R, 813F, and 881R were used for cycle sequencing. Because high polymorphism, pseudogenization, and non-concerted evolution of nrDNA ITS occurs in some flowering plant genera such as *Quercus* (Mayol and Rosselló, 2001) and *Pyrus* (Zheng et al., 2008), we also needed to test whether *A. fulvicoma* was a real interspecific hybrid from *A. eriantha* and *A. cylindrica* using other nuclear genes. Therefore, new primers were specially designed to amplify and sequence four single-copy nuclear genes (SCNGs) from different chromosomes (**Table S2**) according to the sequences of diploid genome of *A. chinensis* cv. Hongyang (Huang et al., 2013).

Polymerase chain reactions were performed in a 50 μl reaction volume containing ~150 ng of total DNA, 5 μl of 10× PCR Buffer (Mg^2+^ free), 6 μl of MgCl_2_ (2.5 mmol·L-1), 8 μl of dNTP Mixture (2.5 mmol·L-1), 3μl of forward primer (10 μmol·L-1), 3μl of reverse primer (10 μmol·L-1), and 2.5 unit of Taq polymerase. The PCR running programs for ITS, *trn*L-*trn*F, *nad2-i3*, and four SCNGs are presented in **Table S3**. PCR products were visualized on 1.0% agarose gels and purified on 3.0% agarose gels using a Gel Extraction System B Kit (BioDev-Tech, Beijing, China). Purified PCR products were sequenced directly by a BigDye Terminator 3.1 Cycle Sequencing Kit (Applied Biosystems, CA, USA) and run in an ABI PRISM 377 DNA Sequencer (Applied Biosystems, CA, USA). For PCR products possessing heterozygous sequences, they were ligated into pMD-18T vector (TaKaRa Co., Dalian, China) and cloned into plasmids. Eight separate clones with correct insert were then screened by comparing restriction fragments for cycle sequencing (Lee et al., 2002; Kim et al., 2008). All sequences generated in this study were submitted to GenBank (accession numbers: MK425065-MK425153 and MK614167-MK614214).

### Phylogenetic Analyses

The sequences of our samples and other sequences obtained from GenBank were assembled by a Seqman II 5.05 (DNAStar, London, UK). The alignment of gene sequences was undertaken by a Clustal X 2.0 (Larkin et al., 2007) and checked manually. All gaps in the alignment were treated as missing data. Maximum likelihood (ML) was performed on three sets of independent sequence data. ML analyses were run in Randomized Accelerated Maximum Likelihood (RAxML) 7.0.4 (Stamatakis, 2006), and the general time reversible (GTR) model and gamma distribution were used for constructing the phylogenetic relationships of the species involved. Support values for ML trees were estimated with 1,000 bootstrap replicates. MEGA 5.1 (Tamura et al., 2011) was used for the input trees. The species of *Clematoclethra* or *Saurauia* were placed as the root of the tree. In each tree, the accession number of the sequence downloaded from GenBank was marked behind the accession name.

### Distribution and Morphology

In view of the hypothesized hybrid origin of *A. fulvicoma* (Liu et al., 2017), we selected *A. fulvicoma* and two putative parental species (*A. eriantha* and *A. cylindrica*) to analyze their natural distributions. The geographic data from specimen records of four main herbaria (IBK, IBSC, KUN, and PE) and previous field investigations (Cui, 1993; Qing and Liu, 2010) were collected to plot the distributions and to check if the two putative parental species overlap geographically. We compared the specimens of *A. fulvicoma* sampled from the type locality, the neighboring areas, and other localities with type specimen, and the specimens identified by Choufen Liang, who edited *Actinidia* of *Flora Republicae Popularis Sinicae* (Liang et al., 1984), were checked to see whether *A. fulvicoma* has intermediate morphological characters of putative parental species, *A. eriantha* and *A. cylindrica*.

## Results

### Morphological Comparison

**Figure 1:**
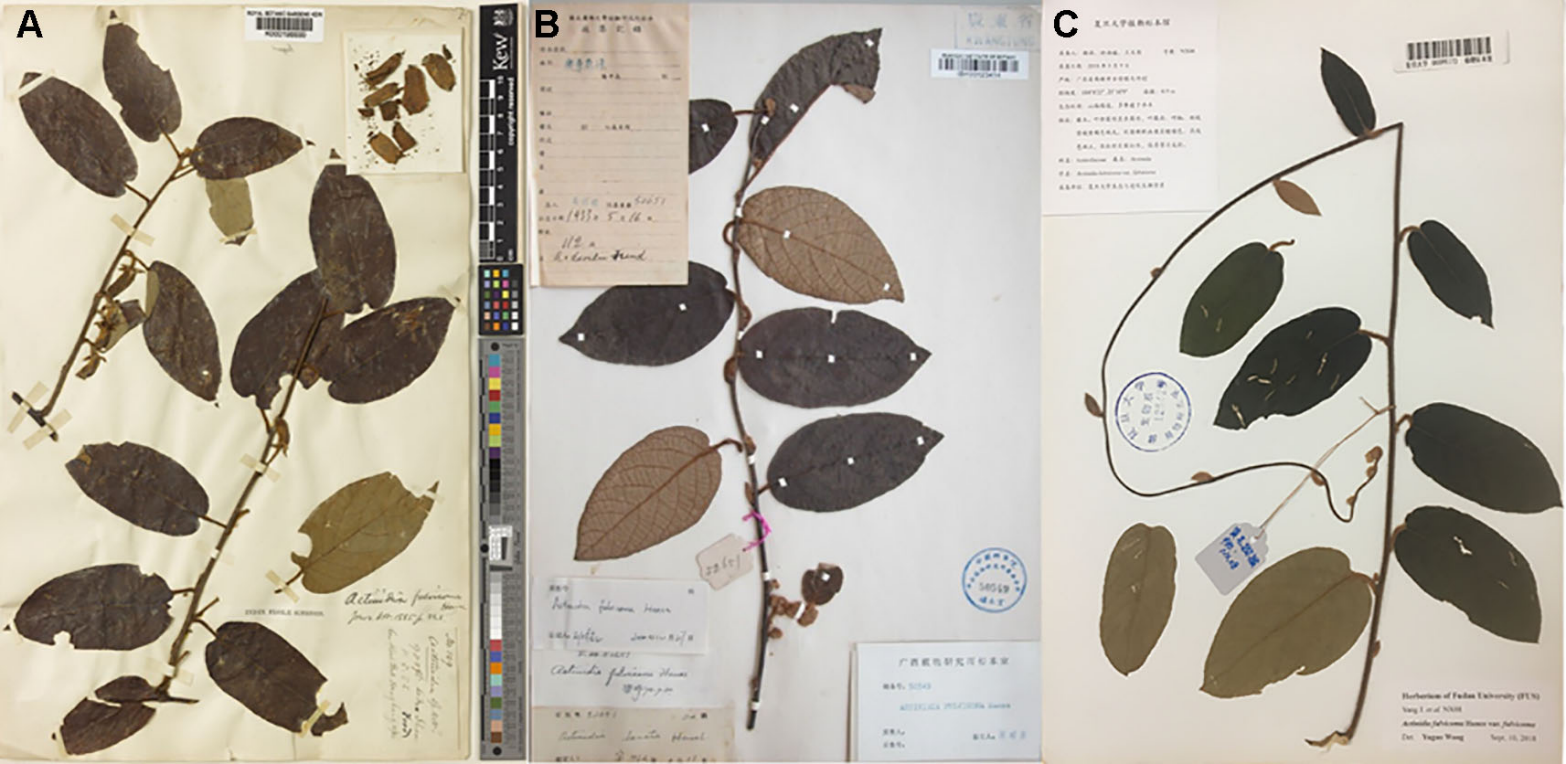
Specimen photographs of *Actinidia fulvicoma*. The type specimen of *A. fulvicoma* deposited in the Herbarium of the Royal Botanic Gardens, Kew **(A)**, the specimens identified by C. Liang **(B)** and collected in this study **(C)**. The leaf blade of *A. fulvicoma* in our specimen has oblong-ovate to suborbicular leaves, the leaf blade is papery, and the leaf venter and young branchlets are covered with dense and stellate tomentum, which shows almost the same morphological characters with the type specimens and the specimen of *A. fulvicoma* identified by C. Liang.

For the type specimen of *A. fulvicoma*, deposited in the Herbarium of the Royal Botanic Gardens, Kew (K) (**Figure 1A**), had a leaf blade that is oblong-ovate and broadly ovate, papery; the branchlets and leaves were abaxially and densely tomentose; the fruit was oblong-ovoid; and persistent sepals reflexed. The specimens identified by C. Liang had similar characters with a little difference on the leaf blade oblong-ovate and they had brownish velutinous (**Figure 1B**). For the samples we collected from the type locality and adjacent areas, the leaf blade was papery, suborbicular to oblong-ovate, fruit narrowly obovate, and speckled, about 0.6–1.5 cm, dark green, persistent sepals reflexed. The morphological characters of these samples fall into the range of populations of *A. fulvicoma*. Although the variations exist in the wild populations, we can still find samples that are very similar to type specimens (**Figure 1C**). Our samples of *A. fulvicoma* from the type locality and adjacent areas were not similar to that of Liu et al. (2017), which voucher picture showed that its morphology was like the two putative parental species, and specially, its fruit was nearly round, close to that of *A. eriantha* [See Figure S1 of Liu et al. (2017)]. Instead, our samples of *A. fulvicoma* showed a high degree of similarity with their ‘*hirsuta*' sample of *A. fulvicoma*, which morphological characters of leaf, flower, and fruit were clearly different to those of *A. eriantha* and *A. cylindrica*.

### Phylogenetic Analyses

#### ITS Data

**Figure 2:**
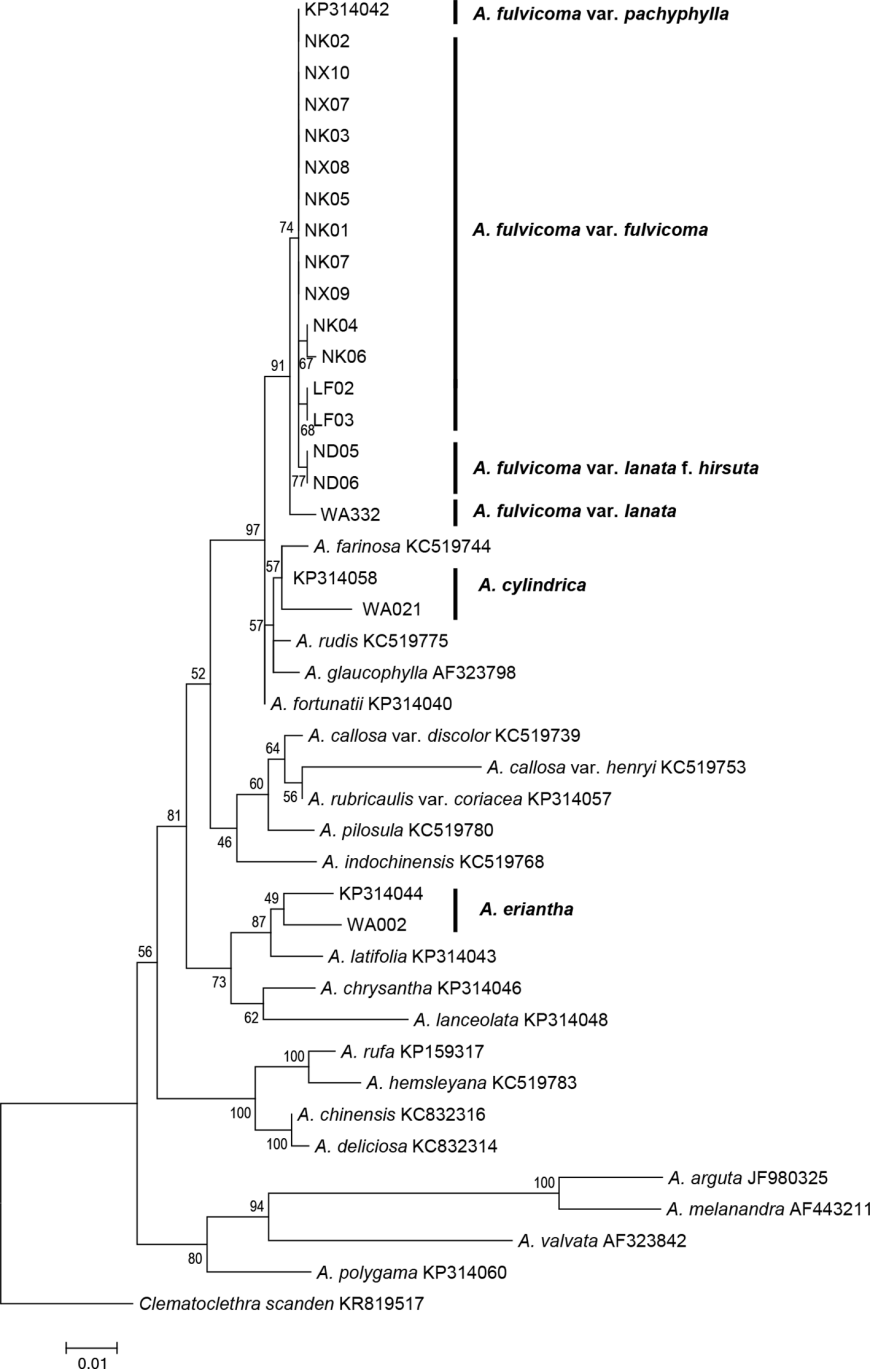
Phylogenetic analysis of *Actinidia* species based on ITS sequences using RAxML. All the samples of *A. fulvicoma* and its varieties form a clade. The ITS tree shows that *A. fulvicoma* is distantly related with the putative parental species *A. eriantha* and *A. cylindrica*, which means that *A. eriantha* and *A. cylindrica* are not the putative parental species of *A. fulvicoma*. Numbers above the branch are bootstrap values result from 1,000 replicates.

The data matrix of 42 sequences from 23 species, including 24 sequences obtained from GenBank and 18 sequences generated in this study, was used to construct an ITS phylogenetic tree. The full-length ITS sequence includes three regions, ITS1, 5.8S, and ITS2. Of the 649 nucleotide sites, 103 sites were parsimony informative after alignment. The GC content of the ITS alignment matrix was 55%. Bootstrap values for maximum likelihood are presented above the branches in **Figure 2**. The phylogenetic tree based on ITS data obtained similar topologies of main clades within *Actinidia* as a previous study (Li et al., 2002). All samples of *A. fulvicoma* and its varieties formed a monophyletic group with 91% maximum likelihood bootstrap (MLBS), and they did not directly cluster with either of the two putative parental species, *A. eriantha* or *A. cylindrica*. *A. eriantha* was sister to *A. latifolia* (87% MLBS), whereas *A. cylindrica* clustered with *A. farinosa* (57% MLBS). *A. fulvicoma* var. *lanata* was sister to other accessions of *A. fulvicoma*. There were seven single nucleotide polymorphisms (SNPs) between var. *lanata* and the common type of *A. fulvicoma* but only one nucleotide difference between var. *lanata* f. *hirsuta* and *A. fulvicoma*. There was no difference between *A. fulvicoma* var. *fulvicoma* and *A. fulvicoma* var. *pachyphylla*. However, there were obvious SNP differences among *A. fulvicoma*, *A. eriantha*, and *A. cylindrica* (**Table 1**). Similar results were obtained by genetic distance analysis: the genetic distance between the population samples of *A. fulvicoma* var. *fulvicoma* and *A. fulvicoma* var. *pachyphylla* was 0–0.00159, which was among the range of all population samples of *A. fulvicoma* (0–0.00479); however, the genetic distance between *A. fulvicoma* and *A. eriantha* and between *A. fulvicoma* and *A. cylindrica* were 0.03998–0.044493 and 0.02238–0.02566, respectively, showing clear differences at the species level. Furthermore, there were more than two ITS haplotypes in the samples from Dayao mountain populations of *A. fulvicoma* isolated by molecular cloning. Phylogenetic analysis shows that they were nested within the same clade of *A. fulvicoma* and its varieties, and no sample clustered with *A. eriantha* or *A. cylindrica* (**Figure S1**).

**Table 1:** The variable sites of nrDNA ITS sequences.

Absolution position										1	1	1	2	2	2	2	2	2	2	4	4	4	4	5	5	5	5	5	5	5	5	5	5	5	5	6	6
	2	2	3	7	3	8	8	8	9	0	1	9	0	0	2	2	3	4	4	3	5	8	8	1	1	3	5	6	7	7	7	8	8	9	9	0	2
	1	5	9	3	1	5	6	9	6	6	9	2	2	3	1	2	9	1	6	8	3	4	5	7	8	5	0	7	3	5	8	2	7	0	9	1	7
*A. fulvicoma* var. *fulvicoma*																																					
NX07	T	C	T	T	C	T	G	T	T	G	T	T	A	T	T	C	C	T	G	G	C	T	T	-	-	A	C	T	C	-	C	G	T	T	T	T	T
NX08	T	C	T	T	C	T	G	T	T	G	T	T	A	T	T	C	C	T	G	G	C	T	T	-	-	A	C	T	C	-	C	G	T	T	T	T	-
NX09	T	C	T	T	C	T	G	T	T	G	T	T	A	T	T	C	C	T	G	G	C	T	T	-	-	A	C	T	C	-	C	G	T	T	T	T	T
NX10	T	C	T	T	C	T	G	T	T	G	T	T	A	T	T	C	C	T	G	G	C	T	T	-	-	A	C	T	C	-	C	G	T	T	T	T	T
NK01	T	C	T	T	C	T	G	T	T	G	T	T	A	T	T	C	C	T	G	G	C	T	T	-	-	A	C	T	C	-	C	G	T	T	T	T	T
NK02	T	C	T	T	C	T	G	T	T	G	T	T	A	T	T	C	C	T	G	G	C	T	T	-	-	A	C	T	C	-	C	G	T	T	T	T	T
NK03	T	C	T	T	C	T	G	T	T	G	T	T	A	T	T	C	C	T	G	G	C	T	T	-	-	A	C	T	C	-	C	G	T	T	T	T	-
NK04	T	T	T	T	C	T	G	T	T	G	T	T	A	T	T	C	C	T	G	G	C	T	T	-	-	A	C	T	C	-	C	G	T	T	T	T	T
NK05	T	C	T	T	C	T	G	T	T	G	T	T	A	T	T	C	C	T	G	G	C	T	T	-	-	A	C	T	C	-	C	G	T	T	T	T	T
NK06	T	T	T	T	C	T	G	T	T	G	T	T	A	T	T	C	C	T	G	G	C	T	T	-	-	A	C	T	C	-	C	G	T	T	T	T	G
NK07	T	C	T	T	C	T	G	T	T	G	T	T	A	T	T	C	C	T	G	G	C	T	T	-	-	A	C	T	C	-	C	G	T	T	T	T	T
LF02	T	C	T	T	C	T	G	T	T	G	T	T	A	T	T	C	A	T	G	G	C	T	T	-	-	A	C	T	C	-	C	G	T	T	T	T	T
LF03	T	C	T	T	C	T	G	T	T	G	T	T	A	T	T	C	A	T	G	G	C	T	T	-	-	A	C	T	C	-	C	G	T	T	T	T	T
*A. fulvicoma* var. *pachyphylla*																																					
KP314042	T	C	T	T	C	T	G	T	T	G	T	T	A	T	T	C	C	T	G	G	C	T	T	-	-	A	C	T	C	-	C	G	T	T	T	T	T
*A. fulvicoma* var. *lanata*																																					
WA332	T	C	A	T	C	T	G	-	T	C	T	T	A	C	T	C	C	T	G	G	C	T	T	T	T	A	T	T	C	-	C	G	T	T	T	T	T
*A. fulvicoma* var. *lanata* f. *hisuta*																																					
ND05	T	C	T	T	C	T	G	T	T	G	T	T	A	T	T	C	C	T	G	G	C	T	T	-	-	A	C	T	C	-	T	G	T	T	T	T	T
ND06	T	C	T	T	C	T	G	T	T	G	T	T	A	T	T	C	C	T	G	G	C	T	T	-	-	A	C	T	C	-	T	G	T	T	T	T	T
*A. eriantha*																																					
KP314044	C	C	T	C	-	C	C	C	C	C	C	T	T	G	C	T	C	C	A	A	T	C	C	-	-	G	C	C	T	C	T	A	T	C	C	G	T
WA002	C	C	T	C	-	C	C	C	C	C	C	T	T	T	T	T	C	T	A	A	T	C	C	-	-	G	C	C	T	C	T	A	T	C	T	G	T
*A. cylindrica*																																					
KP314058	T	C	T	T	C	T	G	T	T	C	T	C	A	T	T	C	C	T	A	G	T	T	T	-	-	A	C	T	C	-	C	G	T	T	T	T	T
WA021	T	C	T	T	C	T	G	T	T	C	T	C	A	T	T	C	C	T	A	G	T	T	T	-	-	A	C	T	C	-	C	G	T	T	T	T	T

#### Mitochondrial and Chloroplast Data

The alignment of mtDNA *nad2-i3* is 1687 bp, and only 14 were parsimony informative characters with an insertion/deletion (InDel). The *nad2-i3* gene data contains 35 sequences from 22 species, an all sequences were generated in this study. The GC content of the *nad2-i3* alignment matrix was 47.5%. Despite the poor phylogenetic resolution (MLBS < 50%), either *A. eriantha* or *A. cylindrica* still formed a monophyly (**Figure S2**). There were four phylogenetic informative characters and a 5-bp InDel among *A. fulvicoma*, *A. eriantha*, and *A. cylindrica*. As shown in **Table 2**, there were three distinct SNP sites and a 4-bp InDel difference between the *nad2-i3* nucleotide sequence of *A. cylindrica* and *A. fulvicoma*. *A. eriantha* showed a nucleotide sequence that was similar to seven individuals of *A. fulvicoma*, with the exception of a difference in A/G at site 1032.

**Table 2:** The informative characters of *nad2i3* and *trn*L-*trn*F sequences.

Absolute position	mtDNA *nad2i3*								cpDNA *trn*L-*trn*F															
								1																
	3	5	6	6	6	6	7	0		2	2	2	3	3	6	7	7	7	7	7	7	7	8	8
	2	3	0	0	0	1	1	1	5	7	8	8	1	4	8	0	0	1	2	2	2	2	0	2
	9	7	7	8	9	0	0	7	1	1	4	5	2	0	7	3	8	9	0	1	2	3	8	6
*A. fulvicoma*																								
NK01	C	G	A	G	A	C	A	A	C	C	-	-	G	A	C	G	C	-	-	-	-	-	C	G
NK02	C	G	A	G	A	C	A	A	C	C	-	-	G	A	C	G	C	-	-	-	-	-	C	G
NK03	C	G	A	G	A	C	A	A	C	C	-	-	G	A	C	G	C	-	-	-	-	-	C	G
NK04	C	G	A	G	A	C	A	A	C	C	-	-	G	A	C	G	C	-	-	-	-	-	C	G
NK05	C	G	A	G	A	C	A	A	C	C	-	-	G	A	C	G	C	-	-	-	-	-	C	G
NK06	C	G	A	G	A	C	A	A	C	C	-	-	G	A	C	G	C	-	-	-	-	-	C	G
NK07	C	G	A	G	A	C	A	A	C	C	-	-	G	A	C	G	C	-	-	-	-	-	C	G
NX07	C	G	A	G	A	C	A	A	C	C	-	-	G	A	C	G	C	-	-	-	-	-	C	G
NX08	C	G	A	G	A	C	A	A	C	C	-	-	G	A	C	G	C	-	-	-	-	-	C	G
NX09	C	G	A	G	A	C	A	A	C	C	-	-	G	A	C	G	C	-	-	-	-	-	C	G
NX10	C	G	A	G	A	C	A	A	C	C	-	-	G	A	C	G	C	-	-	-	-	-	C	G
NX10	C	G	A	G	A	C	A	A	C	C	-	-	G	A	C	G	C	-	-	-	-	-	C	G
*A. cylindrica*																								
OS17	A	T	-	-	-	-	C	A	C	T	-	T	G	A	C	G	C	-	-	-	-	-	C	G
WA021	A	T	-	-	-	-	C	A	C	T	-	T	G	A	C	G	C	-	-	-	-	-	C	G
*A. eriantha*																								
WA002	C	G	A	G	A	C	A	G	T	T	T	T	A	G	A	A	T	G	A	T	T	C	A	A
WA018	C	G	A	G	A	C	A	G	T	T	T	T	A	G	A	A	T	G	A	T	T	C	A	A
WA027	C	G	A	G	A	C	A	G	T	T	T	T	A	G	A	A	T	G	A	T	T	C	A	A

The alignment length of cpDNA *trn*L*-trn*F was 926 bp; it contained nine parsimony informative characters as well as two InDels. The *trn*L*-trn*F gene data include 33 sequences from 15 species, and 11 sequences were obtained from GenBank and 22 sequences were generated in this study. The GC content of the *trn*L*-trn*F alignment matrix was 33.9%. In the *trn*L*-trn*F tree, *A. fulvicoma* clustered with *A. cylindrica*, but the accessions of *A. fulvicoma* did not form a monophyly (**Figure S3**). There was one C/T mutations at site 271 and a T insert at sites 284–285 between *A. fulvicoma* and *A. cylindrica*, but there were also some sequence differences between *A. fulvicoma* and *A. eriantha* that involved a 2-bp InDel and a 5-bp InDel at sites 284–285 and 719–723, respectively, and additional ten SNP sites. The nucleotide variation sites among *Actinidia* species in this study are shown in **Table 2**.

#### The Data of SCNGs

**Figure 3:**
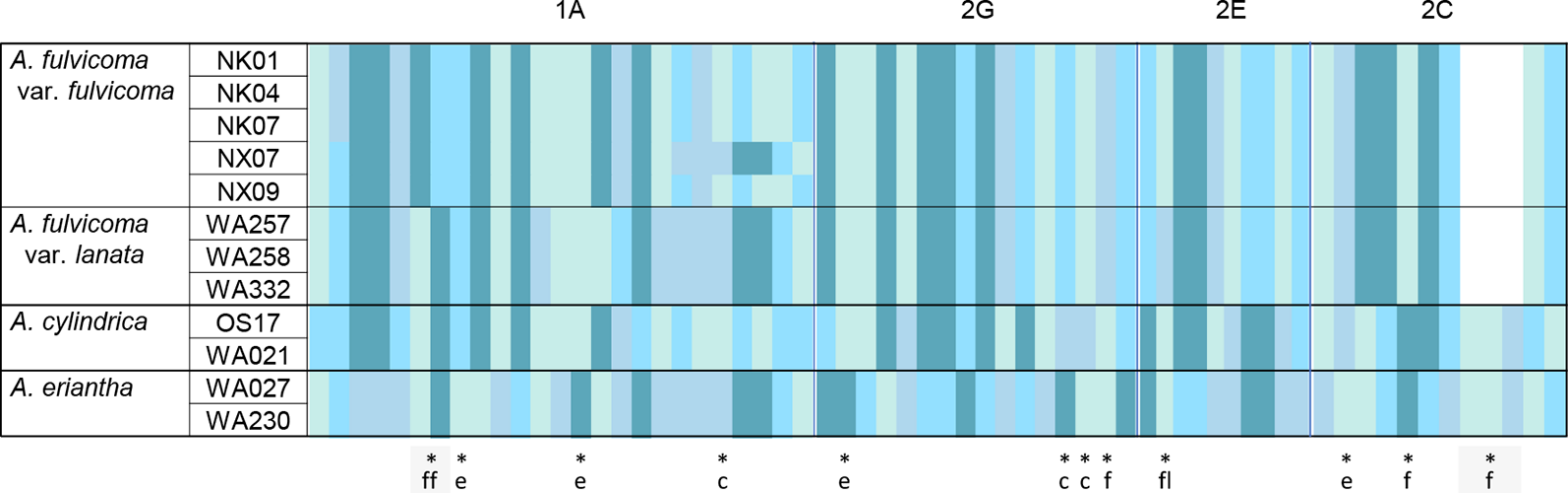
The informative characters from sequences of four SCNGs (1A, 2G, 2E, and 2C) in *A. fulvicoma*, *A. eriantha*, and *A. cylindrica*. *A. fulvicoma* contained no genetic component from the two putative parents identified by Liu et al. (2017), *A. eriantha*, and *A. cylindrica*, suggesting that *A. fulvicoma* is an independent species, rather than a hybrid species. Gap is indicated in white, and four types of nucleotides are indicated in different colors. The corresponding species (or variety)-specific amino acid sites are marked with an asterisk and the abbreviation of the taxon name (c, *A. cylindrica*; e, *A. eriantha*; f, *A. fulvicoma*; ff, *A. fulvicoma* var. *fulvicoma*, and fl, *A. fulvicoma* var. *lanata*).

The four aligned SCNGs (1A, 2G, 2E, and 2C) in *A. fulvicoma* var. *fulvicoma*, *A. fulvicoma* var. *lanata*, *A. cylindrica*, and *A. eriantha* were 411 bp, 565 bp, 739 bp, and 532 bp, respectively. Among these SCNGs, there were 25, 16, and 10 informative characters in 1A, 2G, and 2E, respectively, and 9 informative characters with a 3-bp InDel in 2C. In each SCNG, *A. fulvicoma* var. *fulvicoma* showed obvious informative character differences in various numbers of SNPs or InDel with either *A. cylindrica* or *A. eriantha* (**Figure 3**, **Table S3**). Similar to ITS, the four SCNGs suggested that neither *A. cylindrica* nor *A. eriantha* was the parent species of *A. fulvicoma* despite it being a hybrid species. Although most informative sites of these SNPs were synonymous mutations, the presence of species-specific non-synonymous amino acid sites in these functional genes supported that *A. fulvicoma* was an independent species different from *A. cylindrica* and *A. eriantha*. There was no sequence additivity from these two putative parental species found in *A. fulvicoma*. Similar non-synonymous amino acid mutations could even distinguish the two varieties of *A. fulvicoma*, var. *fulvicoma* and var. *lanata*.

### Distribution of *A. fulvicoma* and Related Species

**Figure 4:**
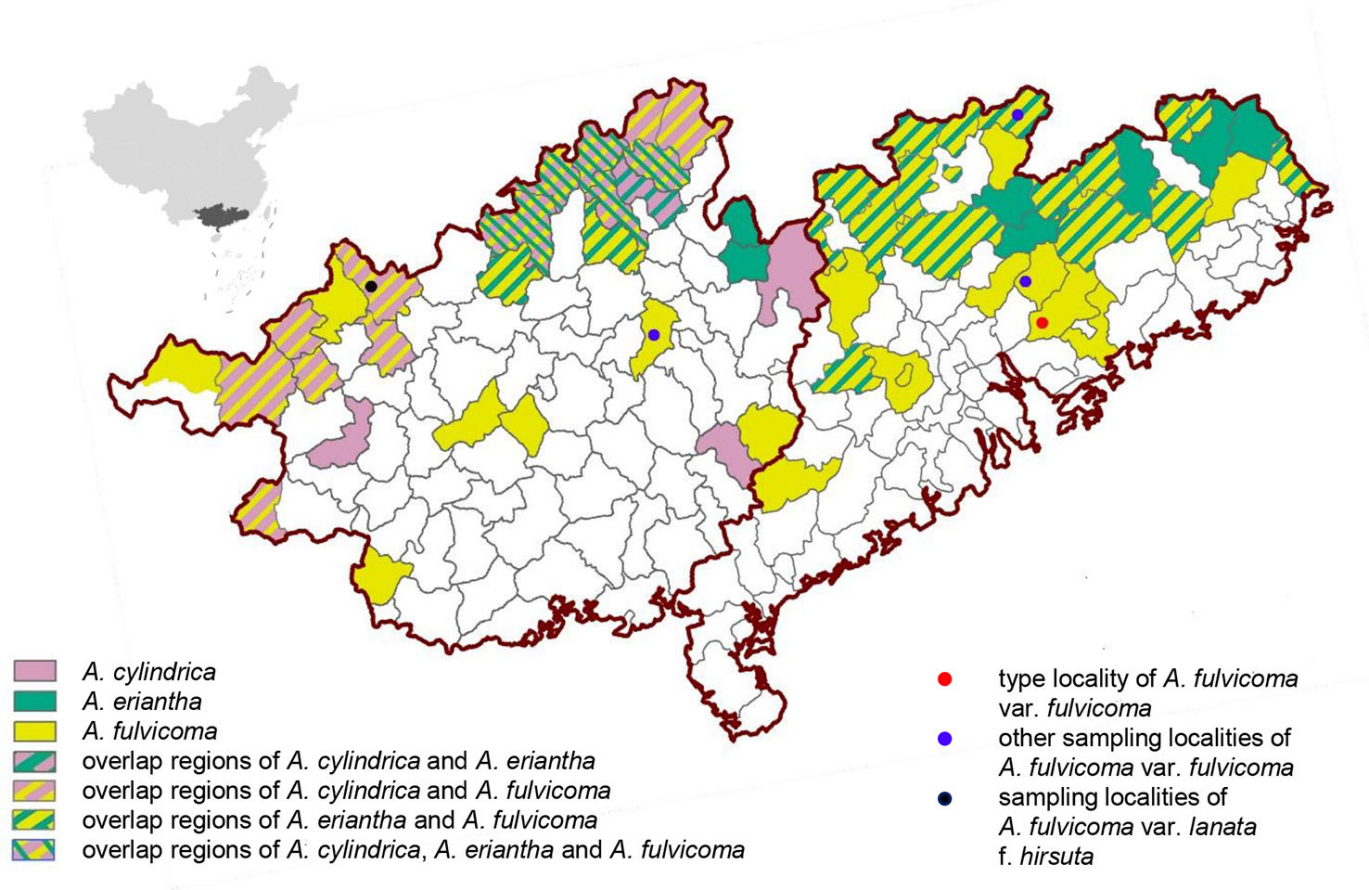
The geographical distribution map of *A. cylindrica*, *A. eriantha*, and *A. fulvicoma* in Guangxi and Guangdong in China. The figure on the top left corner is a map of China showing the locations of Guangxi (left) and Guangdong (right) within China (black area). All counties with distribution records are shown in the map. The distribution areas of the three kiwifruit species are indicated by different colors (*A. cylindrica*, pink; *A. eriantha*, green; and *A. fulvicoma*, yellow), and their overlapping regions are shown by different types of stripes. The four sampling sites from different populations of *A. fulvicoma* are indicated by different colored dots.

*A. fulvicoma* is widely distributed in southern China (e.g. Guangdong, Guangxi, Jiangxi, and Hunan) and its type locality is Mount Luofu, Guangdong. *A. cylindrica* is endemic to north Guangxi, only existing in some counties, whereas *A. eriantha* is widely distributed in several provinces of China, such as Zhejiang, Fujian, Guangdong, Guangxi, and Jiangxi, according to previous studies (Cui, 1993; Qing and Liu, 2010). To investigate the distribution characteristics of *A. fulvicoma* and the two putative parental species, the distribution maps of three species are drawn in the main geographical overlapping areas of Guangdong and Guangxi (**Figure 4**). The distribution of *A. fulvicoma*, *A. cylindrica*, and *A. eriantha* is represented by yellow, pink, and green, respectively. The two putative parental species documented by Liu et al. (2017) are not sympatric in the type locality of *A. fulvicoma*, Mount Luofu, or even in the Guangdong province. Only a few overlapping areas of *A. cylindrica* and *A. eriantha* exist in the north of Guangxi.

## Discussion

### Multiple Lines of Evidence Reveal That *A. fulvicoma* Is Not a Hybrid Species

Despite high similarity with one of putative parents identified by Liu et al. (2017), *A. cylindrica* or *A. eriantha*, in *trn*L-*trn*F and *nad2-i3* sequences, the samples of *A. fulvicoma* possessed a unique haplotype that is distinct from those of the two putative parental species. Our results did not support the result of Liu et al. (2017), which suggested that *A. cylindrica* was the female parent of *A. fulvicoma* and *A. eriantha* the male parent. According to the inference of Liu et al. (2017), *A. fulvicoma* should have had *A. cylindrica*-type mtDNA *nad2-i3* and *A. eriantha*-type cpDNA *trn*L-*trn*F, but this was not the case. A more critical piece of evidence from ITS phylogenetic analysis showed that no sequence of these two putative parental species and any other kiwifruit species existed in *A. fulvicoma*, let alone molecular additivities of informative characters of ITS sequences. *A. fulvicoma* occupied a monophyletic clade, which is far from either *A. cylindrica* or *A. eriantha*. Although ITS sequences in some flowering plant genera (e.g. *Pyrus* and *Quercus*) experience non-concerted evolution, the situation in *A. fulvicoma* is quite different. It has been proven that the high ITS polymorphism of Dayao Mountain population originated from intraspecific hybridization, being entirely unrelated to *A. cylindrica* and *A. eriantha*. All samples of *A. fulvicoma* from different populations form a monophyly. The informative characters from sequences of four SCNGs also showed that *A. fulvicoma* was an independent species that contains no genetic component from these two putative parental species. Thus, our data did not support that *A. fulvicoma* is a hybrid of *A. cylindrica* and *A. eriantha*.

Morphologically, the flower and fruit characteristics of Liu et al. (2017)'s sample were completely inconsistent with the original description of *A. fulvicoma*. Unlike their sample, all the samples that we collected from type locality and other places fell into the normal variation range of A. *fulvicoma*, and they did not present typical intermediate characteristics between *A. eriantha* and *A. cylindrica*. These morphological comparisons indicated that an incorrect sample led to error conclusion for the hybrid origin of *A. fulvicoma* in the study of Liu et al. (2017).

Furthermore, current distribution information also does not support the hybrid origin of *A. fulvicoma*. For the two putative parental species of Liu et al. (2017), *A. cylindrica* is endemic to Guangxi while *A. eriantha* is widely distributed in Fujian, Hunan, Guizhou, Guangdong, Guangxi, etc. However, type specimens of *A. fulvicoma* collected from Mount Luofu in Guangdong were outside of the natural distribution area of *A. cylindrica*, and, thus, they were impossible to hybridize with *A. eriantha* in the type locality. Although hybridization between the putative parental species might have occurred, as the result of sporadic contact long ago, for example, leaving no clear traces at present, ecological niche modeling showed that the decrease in the potential contact regions of two putative parental species since the Last Glacial Maximum (LGM) did not occur in *Actinidia* backbone taxa (Figure S20 in Liu et al., 2017). In general, Guangdong and Guangxi of South China are very suitable for the growth of different kiwifruit species. The extremely narrow distribution of *A. cylindrica* should not be the result of its area reduction or replacement of *A. fulvicoma*. Currently, there is no overlapping between *A. cylindrica* and *A. eriantha* in the entire Guangdong province, including the type locality of *A. fulvicoma*, neighboring regions, and most distribution areas in Guangxi. More importantly, none of our *A. fulvicoma* samples were of a hybrid origin between these two species.

Species misidentification and sampling error can lead to wrong conclusions of hybrid origins. In this study, multiple lines of evidence have confirmed that *A. fulvicoma* is an independent species, rather than a hybrid species. The sample of Liu et al. (2017) should be an interspecific hybrid between *A. cylindrica* and *A. eriantha*, probably collected from their overlapping regions in the north of Guangxi (**Figure 3**), but not real *A. fulvicoma*. Even if their sample of *A. fulvicoma* is a hybrid derived from *A. cylindrica* and *A. eriantha*, which is morphologically similar to *A. fulvicoma*, and is found in other localities, this cannot change the fact that *A. fulvicoma* from the type locality is not a hybrid. *A. fulvicoma* is still regarded as an independent species. However, if the samples from the type locality of the original variety of a species are hybrids, the nomenclature of other varieties of the species should be changed, according to the International Code of Botanical Nomenclature (ICBN). This will inevitably give rise to a series of taxonomic problems, but our evidence has shown that it is not the case.

### Niche Theory Involving Natural Hybridization and Species Distribution

The colonization of ecological niches would be a dynamic process, with competition between parental lineages and hybrid populations, and hybridization could widen ecological adaptation (Choler et al., 2004). However, our conclusion that *A. fulvicoma* is a non-hybrid species has explicitly rejected the first prerequisite of the viewpoint of Liu et al. (2017) that the hybrid *A. fulvicoma* should be a winner in the competition for ecological niches in comparison to one of its putative parents, *A. cylindrica*, with a very restricted distribution within its wide geographic range, further suggesting that hybridization can widen ecological adaptation (Choler et al., 2004). In the case of alpine *Carex curvula*, however, Choler et al. (2004) focused on two basically sympatric subspecies, subsp. *rosae* and subsp. *curvula*, and suggested that ecologically marginal populations of each subspecies are mainly composed of individuals with genotypes resulting from introgressive hybridization. The situation is obviously different from that of *A. fulvicoma*: it was identified by Liu et al. (2017) as a hybrid species of two distinct species (*A. eriantha* and *A. cylindrica*), and the distribution area of *A. fulvicoma* is much larger than that of *A. cylindrica* (**Figure 4**). New niche requirements and adaptations have also been found in the studies of invasive species. However, few cases involving species range expansion match the distribution comparison between *A. fulvicoma* and *A. cylindrica*. An exceptional case is *Spartina anglica*, which is originated from allopolyploidization (Baumel et al., 2002; Ainouche et al, 2004) rather than homoploid hybrid speciation, and its invasive success is largely depended on early artificial introduction. We expect to witness a case where the distribution of a species hybridized from two distinct homoploid species is much larger than that of one of its parent species, providing us a new understanding of the role of hybridization on species range expansion. It is not excluded that such examples can be found in other plants, however, whether—and to what extent—hybridization has an effect on the evolution of species ranges requires further study across more diverse taxa (Pfennig et al., 2016). Regardless of whether other hybrid species facilitate range expansion, at least in the case of *A. fulvicoma*, the fact that its geographical range is much larger than that of *A. cylindrica* is not caused by hybridization or introgression. Therefore, new evidence is needed to re-evaluate whether the hybrid species in kiwifruit occupy the large regions than their parent species because *A. fulvicoma* is identified by Liu et al. (2017) as the only interspecific hybrid species in *Actinidia* which distribution range is far larger than one of its parent species.

### The Implication for Future Practice on Genomic Studies of Hybrid Speciation

Using conventional approaches, this study has provided unambiguous evidence that rejects the hybrid speciation hypothesis of a crucial kiwifruit species, *A. fulvicoma*, despite genomic evidence of frequent interspecific gene flow in kiwifruit. Considering that interspecific hybridization in flowering plants is relatively common (Rieseberg et al., 1995; Arnold, 1997) but homoploid hybrid speciation with strict standard is rare (Schumer et al., 2014), our case study of testing the hybrid origin hypothesis has highlighted that correct species identification is the premise and basis of hybrid speciation studies. In the taxonomic practice, similar incorrect species identification can be completely avoided but may still happen in the studies of other organisms. Observing hybrid ancestry in the genome of a few samples only provides direct evidence that they have an admixed genome (or even a genome of hybrid origin) but does not necessarily arrive at the optimistic conclusion of hybrid speciation (Schumer et al., 2018). Rigorous researchers tend to delimit the species boundaries using different approaches before identifying hybrid speciation events (e.g. Dejaco et al., 2016). In contrast, if an interspecific hybrid cannot be verified well, such results will mislead further studies of hybrid speciation. Therefore, our empirical approaches, presented here, have demonstrated the importance of the integration of morphological characters from type and non-type specimens, molecular data of multiple genes, and geographic information for correct species identification of putative interspecific hybrids. For the taxa involving frequent interspecific hybridization, we expect that extensive surveys of the putative hybrid and its possible parents, and multiple lines of evidence with strict criteria can be applicable to investigate a wider range of organisms, avoiding species misidentification and unreasonable judgment of hybrid speciation in further genomic analyses.

## Data Availability Statement

The datasets generated for this study can be found in GenBank, MK425065-MK425153; MK614167-MK614214.

## Author Contributions

JieY, HX, and YW collected plant materials. JieY performed the experiments, analyzed data, and wrote the first version of the manuscript. WF, HX, ZS, WZ, and JiY analyzed the results and revised the manuscript. YW designed experiments, supervised the study, and co-wrote and revised the manuscript. All authors contributed to and approved the final manuscript.

## Funding

This research was financially supported by the National Natural Sciences Foundation grants of China (31870202, 31370248) and the National Basic Research Program of China (973 programs, No. 2014CB954100).

## Conflict of Interest

The authors declare that the research was conducted in the absence of any commercial or financial relationships that could be construed as a potential conflict of interest.
