# Supplementary material for: An Empirical Analysis Rejects the Hybrid Speciation Hypothesis of a Crucial Kiwifruit Species, Despite Genomic Evidence of Frequent Interspecific Gene Flow in the Genus
Source: Front Genet. 2020 Feb 4;10:1250. doi: 10.3389/fgene.2019.01250 (PMC7011101; doi:10.3389/fgene.2019.01250)
Supplement: Supplementary file 1 [file DataSheet_1.pdf]

Supplemental Information for:

An Empirical Analysis Rejects the Hybrid Speciation Hypothesis of a Crucial Kiwifruit Species, Despite Genomic Evidence of Frequent Interspecific Gene Flow in the Genus

Jie Yang, Weirui Fu, Haoming Xu, Zhiping Song, Wenju Zhang, Ji Yang & Yuguo Wang

The following Supporting Information is available for this article:

Figure S1 | Phylogenetic tree of *Actinidia* based on ITS data with ML method involving the sequences isolated by molecular cloning from *A. fulvicoma*. Only one cloning sequence of identical haplotype is kept in ITS tree, and the different haplotypes of each individual are represented by H1~H4.

Figure S2 | Phylogenetic tree of *Actinidia* based on mtDNA *nad2-i3* data with ML method.

Figure S3 | Phylogenetic tree of *Actinidia* based on cpDNA *trnL-trnF* data with ML method.

Table S1 | The samples of *A. fulvicoma*, *A. cylindrica* and *A. eriantha* in this study.

Table S2 | PCR amplification programs of genes in this study.

Table S3 | The informative characters and InDels of the sequences of four single copy nuclear genes (1A, 2G, 2E and 2C) in *A. fulvicoma*, *A. eriantha* and *A. cylindrica*.

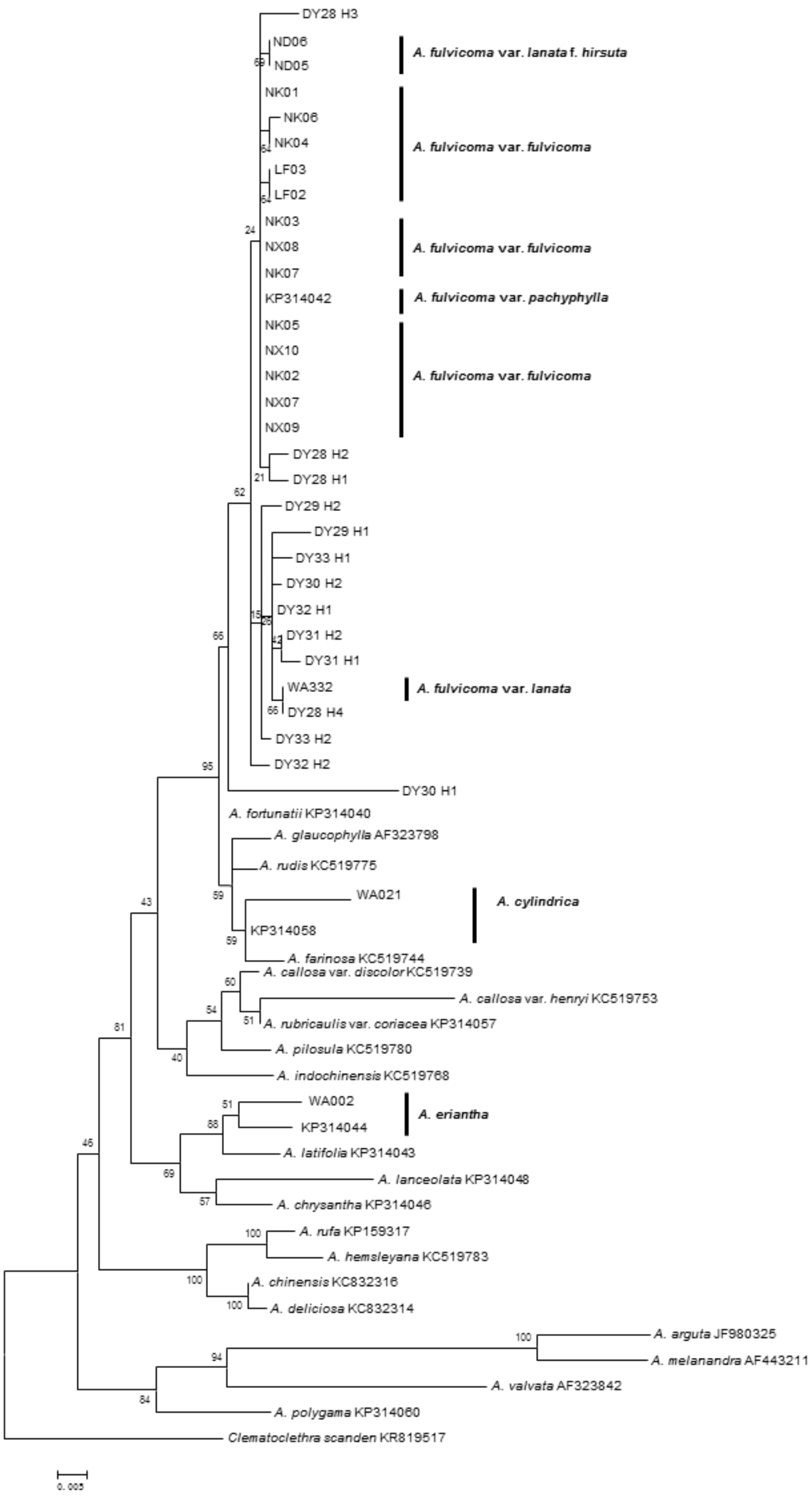

FIGURE S1 Phylogenetic tree of *Actinidia* based on ITS data with ML method involving the sequences isolated by molecular cloning from *A. fulvicoma.* Only one cloning sequence of identical haplotype is kept in ITS tree, and the different haplotypes of every individuals from Dayao mountain are represented by H1~H4.

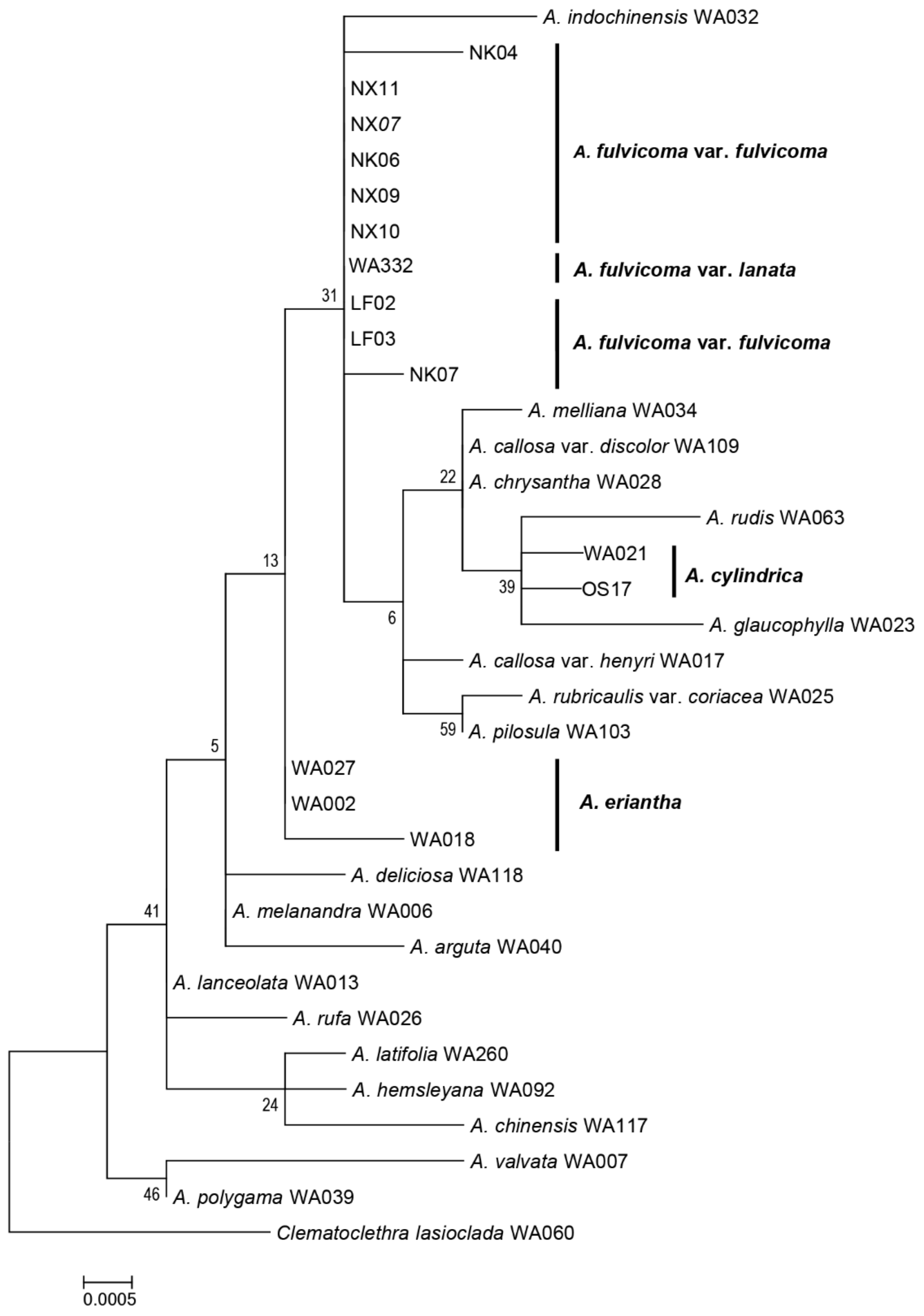

FIGURE S2 Phylogenetic tree of *Actinidia* based on mtDNA *nad2-i3* data with ML method.

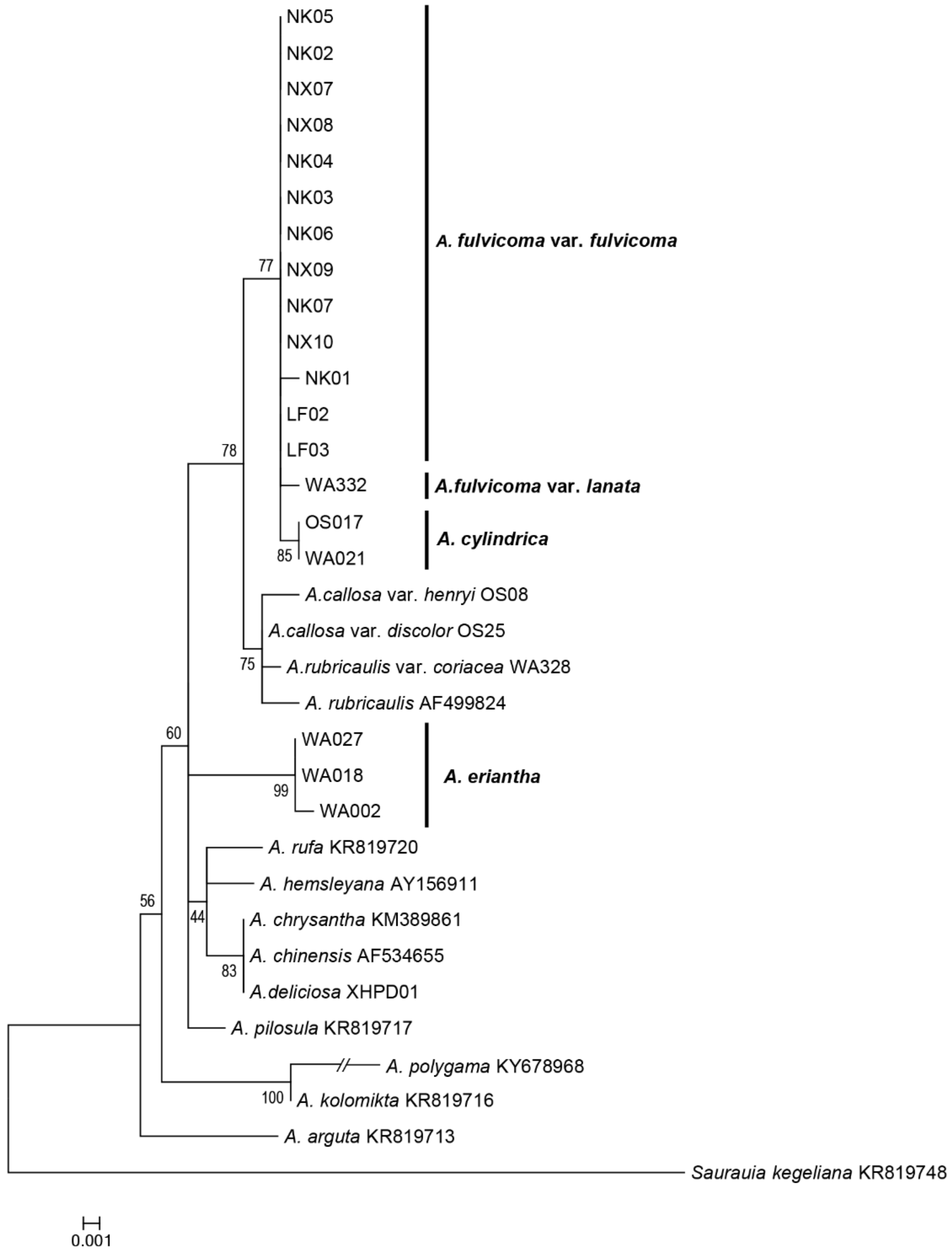

FIGURE S3 Phylogenetic tree of *Actinidia* based on cpDNA *trn*L-*trn*F data with ML method.

Table S1 | The samples of *A. fulvicoma, A. cylindrica* and *A. eriantha* in this study.

Taxa	Accession No.	Site
A. fulvicoma	DY28-DY33;	Dayao mountain, Jinxiu county, Guangxi
	NX07-NX11	Daping village, Nanxiong city, Guangdong
	NK01-NK07	Mt. Nankun, Longmen county, Guangdong
	LF02, LF03	Mt. Luofu, Boluo county, Guangdong
A. fulvicoma var. *lanata*	WA332	Guilin Botanic Garden, Guilin, Guangxi
f. *hirsuta*	ND05, ND06	Nandan county, Guangxi
A. cylindrica	WA021	Guilin Botanic Garden, Guilin, Guangxi
	OS17	Lingui county, Guilin, Guangxi
A. eriantha	WA002, WA018, WA027, WA230	Guilin Botanic Garden, Guilin, Guangxi

Table S2 | PCR amplification programs of genes in this study.

Gene	primers	Denaturation time	Annealing time	Extension time	Annealing Temperature
ITS	ITS4/ITS5	30s	45s	60s	54℃
nad2-i3	2F/1277R	30s	60s	90s	51℃
	2F/881R	30s	45s	60s	53℃
	769F/1R	30s	45s	60s	51℃
	813F/1R	30s	45s	60s	52℃
*trn*L-*trn*F	c/f	30s	60s	90s	51℃

Table S3 | The informative characters and indels of four single copy nuclear genes (1A, 2G, 2E, 2C) sequences of *A. fulvicoma*, *A. eriantha* and *A. cylindrica*.

	1A	2G	2E	2C
**Absolution position**	2222333333344444445555555<br>3334111156922346791345779<br>2672034859206601720895076	1122233333345567<br>8903900011983800<br>0574503689731475	2233555567<br>1602488913<br>7537629455	122224566666<br>903360411133<br>260135945603
A. fulvicoma var. *fulvicoma*				
NK01	AGCCGCTTCACAAACGCATGATAAT	CAACACCTCGTATAGT	TACCGATTAT	AGCCACT---AT
NK04	AGCCGCTTCACAAACGCATGATAAT	CAACACCTCGTATAGT	TACCGATTAT	AGCCACT---AT
NK07	AGCCGCTTCACAAACGCATGATAAT	CAACACCTCGTATAGT	TACCGATTAT	AGCCACT---AT
NX07	ATCCGCTTCACAAACGCAGGGCCTA	CAACACCTCGTATAGT	TACCGATTAT	AGCCACT---AT
NK09	ATCCGCTTCACAAACGCATGATAAT	CAACACCTCGTATAGT	TACCGATTAT	AGCCACT---AT
A. fulvicoma var. *lanata*				
WA257	ATCCGACTCACGAAATCGGGGCCTA	CAACACCTCGTATAGT	TGCCGATTAT	AGCCACT---AT
WA258	ATCCGACTCACGAAATCGGGGCCTA	CAACACCTCGTATAGT	TGCCGATTAT	AGCCACT---AT
WA332	ATCCGACTCACGAAATCGGGGCCTA	CAACACCTCGTATAGT	TGCCGATTAT	AGCCACT---AT
A. eriantha				
WA027	ATGGGACAAGTAGCAGCGGGGCCTA	CCTAGTTCTGTGCAAC	CATTGGCCGG	GAATCTGAAGAT
WA230	ATGGGACAAGTAGCAGCGGGGCCTA	CCTAGTTCTGTGCAAC	CATTGGCCGG	GAATCTGAAGAT
A. cylindrica				
OS17	TTCCTACTCACAAACGTATAATATT	TAACGCCTCACAGGAT	CACCAGCCGT	AGATCCTAAGTA
WA021	TTCCTACTCACAAACGTATAATATT	TAACGCCTCACAGGAT	CACCAGCCGT	AGATCCTAAGTA
